# A linked open data representation of patents registered in the US from 2005–2017

**DOI:** 10.1038/sdata.2018.279

**Published:** 2018-12-04

**Authors:** Mofeed M. Hassan, Amrapali Zaveri, Jens Lehmann

**Affiliations:** 1 Universität Leipzig, Institut für Informatik, AKSW Group, Leipzig, Germany; 2 Institute of Data Science, Maastricht University, Maastricht, The Netherlands; 3 Smart Data Analytics Group at the University of Bonn and Enterprise Information Systems Department, Fraunhofer IAIS, Bonn, Germany

**Keywords:** Technology, Research data, Law, Publishing

## Abstract

Patents are widely used to protect intellectual property and a measure of innovation output. Each year, the USPTO grants over 150,000 patents to individuals and companies all over the world. In fact, there were more than 280,000 patent grants issued in the US in 2015. However, accessing, searching and analyzing those patents is often still cumbersome and inefficient. To overcome those problems, Google indexes patents and converts them to Extensible Markup Language (XML) files using Optical Character Recognition (OCR) techniques. In this article, we take this idea one step further and provide semantically rich, machine-readable patents using the Linked Data principles. We have converted the data spanning 12 years − i.e. 2005−2017 from XML to Resource Description Framework (RDF) format, conforming to the Linked Data principles and made them publicly available for re-use. This data can be integrated with other data sources in order to further simplify use cases such as trend analysis, structured patent search & exploration and societal progress measurements. We describe the conversion, publishing, interlinking process along with several use cases for the USPTO Linked Patent data.

## Background & Summary

A patent is a set of exclusive rights granted to an inventor by a sovereign state for a solution, be it a product or a process, to a particular technological problem^1^. The *United States Patent and Trademark Office* (USPTO) (http://www.uspto.gov/) is part of the *US department of Commerce* and grants patents to businesses and inventors for their inventions in addition to registration of products and intellectual property identification. Each year, the USPTO grants over 150,000 patents to individuals and companies all over the world. As of December 2015, more than 11 million patents have been issued (https://www.uspto.gov/web/offices/ac/ido/oeip/taf/us_stat.htm).

Patents are a form of intellectual property and a measure of innovation output. They cover a broad range of technologies and are a rich source of information. Patents and statistics about them have the following applications:

- facilitate transmission of knowledge from academia to the industry and its application for industrial purposes^2^,
- technological indicator as a sign of transition between science and product, which in turn can be connected to policies of interest such as innovation, economic growth, welfare^3^,
- measure the output of research and development, its productivity, structure and development in a particular technology or industry or specific domains,
- track the level of dissemination of knowledge across technology areas, sectors, firms, countries etc. (http://www.oecd.org/sti/msti.htm),
- statistical indicators of the inventive performance and output of countries, regions, technologies, firms etc.

However, there may be cases when some inventions cannot be patented either because they do not abide by the rules (http://www.uspto.gov/patents-getting-started/general-information-concerning-patents#heading-4), due to ethical reasons, being too abstract, a particular country does not incline towards patenting their innovations or there are different patent regulations (http://www.oecd.org/sti/inno/21682515.pdf), which makes it difficult to compare patenting activities across countries. On the other hand, patents which particularly focus on cross-border tie-ups assist in international comparability, indicate the international flow of knowledge as well as funds for research from the inventor country to the applicant countries (http://www.oecd.org/science/inno/21682515.pdf).

The USPTO patents are accepted in digital format and are filed as PDF documents. Google indexes these patents by using optical character recognition and making them searchable (https://www.google.com/googlebooks/uspto-patents.html). A total of 7 million patents dated from 1790 onwards are available. In these patents, however,, the indexing is not perfect and it is cumbersome to search through the PDF documents due to them not being machine-readable. Google has made all the patents available for download in an Extensible Markup Language (XML) format, albeit only from the years 2002 to present.

In recent years, the Linked Data (LD) paradigm^4^ has emerged as a simple mechanism for employing the Web as a medium for data and knowledge integration where both documents and data are linked. Moreover, the semantics and structure of the underlying data are kept intact, making this the Semantic Web. LD essentially entails a set of best practices for publishing and connecting structure data on the Web, which allows publishing and exchanging information in an interoperable and reusable fashion. Many different communities on the Internet such as geographic, media, life sciences and government have already adopted these LD principles. This is confirmed by the dramatically growing LD Web, where currently more than 50 billion facts are represented (http://lod-cloud.net). These principles are:

- Use Uniform Resource Identifiers (URIs) as names for things.
- Use Hypertext Transfer Protocol (HTTP) URIs so that people can look up those names.
- When someone looks up a URI, provide useful information, using the standards (Resource Description Framework^5^, Simple Protocol and RDF Query Language (SPARQL)^6^, ontologies^7^).
- Include links to other URIs. so that they can discover more things.

Thus, we adopted the Linked Data (LD) principles in order to make this huge amount of valuable information (from 2002 onwards) available in a machine and human-readable format.

Providing the data as LD enables a centralized, structured and heterogeneous source of information. Specifically, the benefits are^8^:

- *A unifying data model.* With globally unique identification of entities expressed using schema to be used in parallel to represent data, the RDF data model enables global data sharing. In contrast, the other methods for publishing data on the Web rely on a wide variety of different data models, and the resulting heterogeneity needs to be bridged in the integration process.
- *A standardized data access mechanism.* LD uses the HTTP protocol, which allows data sources to be accessed using generic data browsers and enables the complete data space to be crawled by search engines. In contrast, Web APIs are accessed using different proprietary interfaces.
- *Hyperlink-based data discovery.* By using Uniform Resource Identifiers (URIs) as global identifiers for entities, LD allows hyperlinks to be set between entities in different data sources. These data links connect all LD into a single global data space and enable applications to discover new data sources at run-time. In contrast, Web APIs as well as data dumps in proprietary formats remain isolated data islands.
- *Self-descriptive data.* LD eases the integration of data from different sources by relying on shared vocabularies, making the definitions of these vocabularies retrievable, and by allowing terms from different vocabularies to be connected to each other by vocabulary links.

Thus, we have converted the data spanning 12 years − i.e. 2005 − 2017 from XML to Resource Description Framework (RDF) format, conforming to the Linked Data principles and made them publicly available for re-use.

## Methods

### An Ontology for Representing Patent Data

W3C defines an ontology as “the terms used to describe and represent an area of knowledge.”^7^. The ontology essentially contains terms and relationships among those terms. Terms are also called classes or concepts. The relationships between these classes can be expressed by using a hierarchy, i.e. superclasses represent higher-level concepts and subclasses represent finer concepts. The finer (sub classes) concepts inherit all the features and attributes that the higher (superclasses) concepts have. Additionally, there is another level of relationship expressed by using a special group of terms called properties. These properties describe various features and attributes of the concepts and are used to associate different classes together. Thus, an ontology defines a set of classes (e.g. “Book”, “Writer”), and their hierarchy, i.e. which class is a subclass of another one (e.g. “Writer” is a subclass of “Person”) along with properties (e.g. a “Book” has an author of type “Writer”).

For the patent data, every Patent is associated with a unique document ID, a title, an abstract, kind of patent, country where it was issued and date of publication. Further, the patent is classified using national and international classifications. The national classification represents the US Patent Classification (http://www.uspto.gov/web/patents/classification/) of the patent while the international classification classifies the patent based on the WIPO International Classifications (http://www.wipo.int/classifications/en/).

**Figure 1:**
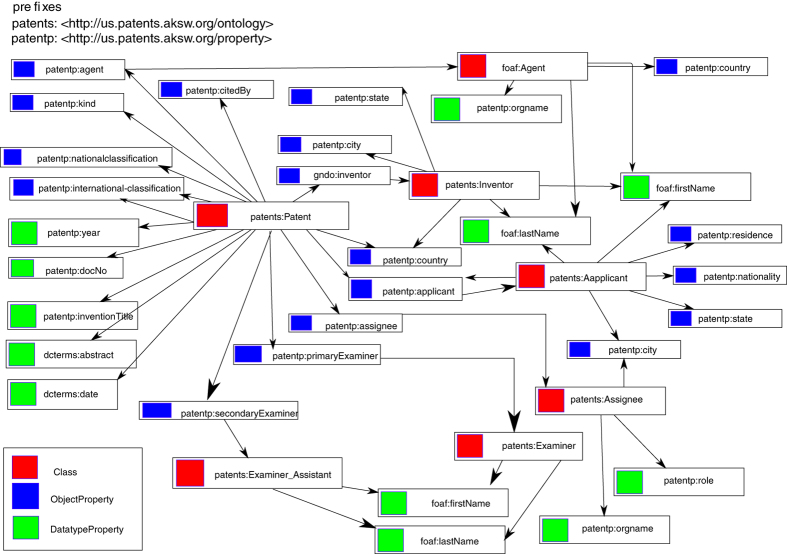
Depiction of the ontology underlying the Linked Patents dataset.

Moreover, each patent is associated with data concerning a set of contributors (to the Patent) and its application. One of the contributors is an Applicant, who applies for the patent. Each applicant’s first name, last name, nationality and residence information are provided accompanied with the city, state and country of origin. Further, the Assignee has the ownership of the patent’s rights and interests, the Examiner and Examiner Assistant examine and analyze the patent and the fulfillment of its application, the Inventor of the patent and the Agent, the one responsible for preparing and persecuting the patent application on behalf of the applicant. Furthermore, each patent file contains data about other Patents that have cited this particular patent. These referenced documents have an associated name and category. This information is modeled as an ontology and is available at https://datahub.io/dataset/linked-uspto-patent-data and depicted in Fig. 1. In accordance with the “Five Stars of Linked Data Vocabulary Reuse”^14^, we have reused vocabularies in our ontology. These vocabularies and their ontologies are listed in Table 1.

### Dataset Conversion and Publishing

The USPTO patents full-text data is available for download in a standard XML format from the years 2005 onwards (http://www.google.com/googlebooks/uspto-patents-grants-text.html). Each week USPTO releases a zipped file of all patents accepted in that week. Each year ca. 52−55 files are published each one containing about 5,000 patents.

In order to convert the XML data to RDF, we utilized the RDF Mapping Language^15^ (RML) (http://semweb.mmlab.be/rml/spec.html). RML is a language for specifying customized mappings from heterogeneous data structures (including Redis Database (RDB), XML, Comma-Separated Value (CSV)) to the RDF data model. We used the already available processor for RML (https://github.com/mmlab/RMLProcessor) to specify the mappings from the XML nodes to the subject, predicate and objects of the RDF format. Two preceding steps to the mapping process were performed. The first step was splitting the XML files of each year into individual patent files. Second, we added XML tags that contain the textual labels of the given alphabet codes for specific elements. For example, for the kinds of patents, the different types are represented using alphabet codes (http://www.uspto.gov/learning-and-resources/support-centers/electronic-business-center/kind-codes-included-uspto-patent). Similarly, the patent classifications, the assignee’s roles, the cities, states and country codes all use different alphabetical codes. Thus, in this step, the actual labels were added so as to transform them into meaningful object values in RDF. All these steps are performed using scripts and Java based programs which are available online at https://github.com/SmartDataAnalytics/linked-uspto-patent-data.

Also, there were differences between the documents in the different years (with different DTDs (Document Type Definitions)), which we modified in the mappings. For example, in the year 2006, the applicant’s address data included the postal code which was not provided in the year 2005. The years 2007 to 2012 had a uniform XML format, while for the years 2013,2014 and 2015 different classifications were used. The size and variation affected the conversion time per year. On average, the conversion of each year took ca. 18 hours and produced total 213,973,552 triples. Box 1 shows an excerpt of a raw XML file of a single patent from the year 2005 and Box 2 showsn an excerpt of the RDF representation of the same patent from the year 2005. An example of the mapping file and the output data is also available on DataHub (https://datahub.io/dataset/linked-uspto-patent-data).

### Dataset Interlinking

Abiding by the fourth Linked Data principle of including links to other related things (using their URIs) when publishing data on the Web, we identified several target links from external datasets. We performed the interlinking using the Link discovery framework for Metric Spaces (LIMES)^16^, which allows a user to specify the source and target properties; metric; relation type as well as the threshold based on which links are discovered between two Linked datasets. The threshold of the similarity measure was set to 0.9 to achieve high quality links. The details of the number of source and target instances as well as the number of accepted links along with the precision and recall for each is recorded in Table 2. In particular, we interlinked the countries between USPTO and DBpedia^17^, LinkedGeoData (LGD) (http://linkedgeodata.org/), World Bank (https://datacatalog.worldbank.org/, https://old.datahub.io/dataset/world-bank-linked-data) as well as EU Patents (available at http://us.patents.aksw.org/sparql). The links were to denote that the URI of the country from one dataset is the *same as* the country from another dataset. Linking the USPTO with both the DBpedia and WorldBank countries, for example, allows to explore and compare related information about that country such as the amount of investment by the government in patent related activities versus the GDP of that country. The links between USPTO and EU Patents ensures a greater coverage of the patents, which helps users (such as potential investors or companies) get an idea of the market trends or competitors in European countries (see examples in the Discussion section for example Application Scenarios).

Additionally cities are interlinked USPTO and other datasets that include DBpedia, EU Patents and LGD. The type of link is also *same as*. This interlinking provides a more specific overview about the patents’ correspondence by sharing the same city. This correspondence can be useful in some social measures, for example, such as measuring the relation between the existence of a university in a city and the number of patents associated to it. Other interlinked entities are state and patent’s invention title between USPTO and EU Patents. The latter interlinking is beneficial to determine the cooperation possibilities between the patent’s inventors and applicants between USA and Europe. Other examples of the benefits of interlinking between datasets is articulated in the Discussion section.

## Data Records

The RDF dataset of the US Patent data is available in the Linked repository (Data Citation 1). The total number of triples produced as a result of the conversion from XML to RDF is 212,234,735. We uploaded all the triples into a Virtuoso (http://virtuoso.openlinksw.com/) triple store, whereby the data can be accessed via a SPARQL endpoint. Links to the dataset, SPARQL endpoint, VoID file and the DataHub entry along with information on the version date, number and licensing are listed in Table 3.

## Technical Validation

In order to ensure that the RDF data can be used reliably in real-world applications, there is a need to ensure that the data is of high quality. Thus, we performed a quality assessment of the conformance of the RML mappings (available at https://datahub.io/dataset/linked-uspto-patent-data) to the RML specifications. This was carried out by using the RML Validator (https://github.com/mmlab/RMLValidator)^18^, a mapping validation tool that is built upon RDFUnit (http://aksw.org/Projects/RDFUnit.html)^19^ and DB2Triples (https://github.com/antidot/db2triples/). RDFUnit is a test-driven evaluation tool that generates a set of test cases to validate the RML mapping. These test cases can be either automatically generated, manually generated, or both. The automatically generated tests, which are used in our assessment, are based on the RDFS/OWL (Web Ontology Language) schema of the assessed dataset. Several methods are implemented to generate such tests. These methods include using RDFS/OWL axioms such as rdfs:domain and rdfs:range, applying schema enrichment techniques, and reusing tests from common vocabularies^19^. After using the RML Validator against the RML Mappings that we generated to convert the Patent data, the output (available at https://datahub.io/dataset/linked-uspto-patent-data) revealed a high compliance of the mapping file to the RML regulations. It marked ‘success’ in all tests except one failure regarding defining the range of some properties. This failure has no effect on the conversion process and it is reported by the validator’s maintainers that the failure may refer to a true error, a waning or a notice.

## Usage Notes

We provide selected application scenarios for using the USPTO Linked Patent data. These use cases are made possible due to the uniform structure of the datasets (i.e. RDF) allowing seamless integration with other relevant datasets, which would otherwise require substantial work with XML data.

### Trend analysis

An advantage of having all the patents in the RDF format is that one can analyze the patents over time i.e. querying for the number of patents in each year. This analysis is possible since the data is stored in a uniform format and thus can be easily aggregated. Using this information, one can measure the growth in number and type of patents over these years, thus allowing governments to analyze the growth in innovation and measure the effect of the investment in research.

**Figure 2:**
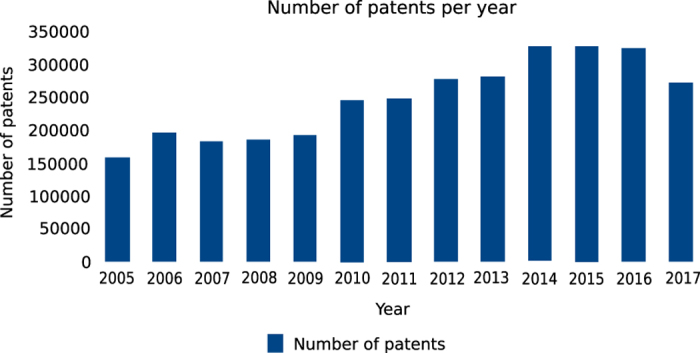
Trend analysis. Shows the growth in total number of patents over the years 2005 to 2017.

Most often, companies also need reports about trends in a particular field over the past 10 years. Moreover, this patent information and analysis can be helpful in identifying new directions of technology that is perhaps currently lacking by querying for patents belonging to a particular field (e.g. biomedical). Box 3 shows the SPARQL query for retrieving the total number of patents per year. This can easily be extended to include specific countries or areas of interest. Fig. 2 shows the trend in total number of patents over the years 2005 to 2017.

### Measuring societal progress

The Linked Patent dataset, when combined with indicators from the World Bank dataset, for instance, can be used to measure specific societal progress indicators. For example, the total number of patents per country can be used as an indicator for innovation, which in turn can serve as a proxy measure for the output of R&D in the form of inventions in that country. This statistic can then be compared with the R&D expenditure (% of GDP) for each country, which can be derived from the World Bank dataset.

Box 4 shows the SPARQL query, which retrieves the country name, total patent count for that country (from the patent dataset) with the amount of R&D expenditure for that country (from the WorldBank dataset). This comparison can thus be used to measure whether the amount invested by a government in R&D is proportional to the innovation churning out of that investment.

### Citation Network

Over the years, there have been studies that have proposed to use patent data to determine the impact of technologies. This is because patents are considered a good proxy for identifying the link and transfer of technological information^9^, especially those that contain more than 90% of the latest technological information in the world^10^. One such method focuses on patent citation networks^11^, where the idea is that a ‘patent cited by subsequent patents has a strong possibility of containing important ideas upon with the later patents are built’^12^.

Thus, patent citations networks help not only identify but also predict emerging technologies and their trends^13^ to illustrate dynamically changing patterns, evolving knowledge flows in a specific technology and to monitor converging technology phenomena^12^. Box 5 shows the SPARQL query to retrieve the number of patent citations according to the patent categories (i.e. based on IPC classes). Further, each individual category can be analyzed to identify local densities corresponding to technological areas, even with temporal changes.

## Additional information

**How to cite this article**: Hassan, M. M. *et al*. A linked open data representation of patents registered in the US from 2005–2017. *Sci. Data*. 5:180279 doi: 10.1038/sdata.2018.279 (2018).

**Publisher’s note**: Springer Nature remains neutral with regard to jurisdictional claims in published maps and institutional affiliations.

## Supplementary Material



## Figures and Tables

**Table 1:** Re-used vocabularies and their classes.

Vocabularies	Classes
**Friend Of A Friend (FOAF)**	*name, firstName, lastName, Assignee,Organization,Agent,Person*
**Resource Description Framework Schema (RDFS)**	*label*
**Dublin Core Terms (DCTERMS)**	*abstract,date*
**Gemeinsame Normdatei Ontology (GNDO)**	*inventor*

**Table 2:** Number of interlinks obtained between USPTO dataset and DBpedia, WorldBank and EU Patents datasets for country, city, state and label data.

Links between	Link type	Source dataset	Source Instances	Target Dataset	Target Instances	No. of Links	Precision	Recall
Countries	owl:sameAs	USPTO	234	DBpedia	1932	351	1	0.87
Countries	owl:sameAs	USPTO	234	World Bank	162	281	1	0.89
Countries	owl:sameAs	USPTO	234	EUPatents	680939	63	1	1
Countries	owl:sameAs	USPTO	234	LGD	228	306	1	1
City	owl:sameAs	USPTO	156761	DBPedia	20886	2331	1	1
City	owl:sameAs	USPTO	156761	EUPatents	37	0	1	1
City	owl:sameAs	USPTO	156761	LGD	11245	3112	1	1
State/Terr.	owl:sameAs	USPTO	59	EUPatents	51	49	1	1
inventionTitle	owl:sameAs	USPTO	3215768	EU Patents	76568	986524	1	1

**Table 3:** Technical details of the USPTO RDF dataset.

**Homepage**	http://www.aksw.org/Projects/USPatents.html
**Namespace**	http://us.patents.aksw.org/
**Total no. of triples**	212,234,735
**SPARQL endpoint**	http://us.patents.aksw.org/sparql
**Version date and number**	January-2018, 2.0
**Licensing**	Attribution-NonCommercial-ShareAlike 4.0 International (CC BY-NC-SA 4.0)
**VoID File**	https://datahub.ckan.io/dataset/476fc1ee-5bd8-4551-bf67-4fe4e7fa349b/resource/05faeb81-4ffc-4033-8093-bd920170d657/download/void.ttl
**figshare Record**	https://doi.org/10.6084/m9.figshare.5970925.v9
**DataHub entry**	https://old.datahub.io/dataset/linked-uspto-patent-data
**RDF Dump**	https://doi.org/10.6084/m9.figshare.5970925.v9

## Excerpt of raw XML file a single patent from the year 2005.

<?xml version="1.0" encoding=" UTF -8"?>

<!DOCTYPE us - patent - grant SYSTEM "us - patent -grant-v40-2004-12-02. dtd" []>

<us - patent - grant lang=" EN">

<us - bibliographic -data -grant>

<publication - reference>

<document -id>

<country>US </country>

<doc - number> D0501074 </doc-number>

<kind>S1</kind>

<date>20050125 </date>

</document -id>

</publication - reference>

<application - reference appl - type="design">

<document -id>

<country>US</country>

<doc - number>29205349</doc -number>

<date>20040512</date>

</document -id>

</application - reference>

<us - application - series -code>29</us - application - series -code>

<classification - locarno>

<edition>7</edition>

<main - classification>0299</main - classification>

</classification - locarno>

<classification - national>

<country>US</country>

<main - classification>D 2971</main - classification>

<further - classification>D2969</further - classification>

</classification - national>

<invention - title id="d0e71">Shoe</invention -title>

<parties>

<applicants>

<applicant sequence="001" app - type="applicant - inventor"designation="us - only">

<addressbook>

<last -name>Kelsey</last -name>

<first -name>Jonathan</first -name>

<address>

<city>London</city>

<country>GB</country>

</address>

</addressbook>

<nationality>

<country>GB</country>

</nationality>

<residence>

<country >GB</country>

</residence>

</applicant>

</applicants>

<agents>

<agent sequence="01"rep - type="attorney">

<addressbook>

<orgname>Fross Zelnick Lehrman & Zissu, P.C.</orgname>

<address>

<country>unknown</country>

</address>

</addressbook>

</agent>

</agents>

</parties>

<assignees>

<assignee>

<addressbook>

<orgname>J. Choo (Jersey) Limited</orgname>

<role>03</role>

<address>

<city>London</city>

<country>GB</country>

</address>

</addressbook>

</assignee>

</assignees>

<examiners>

<primary - examiner>

<last -name>Simone</last -name>

<first -name>Dominic</first -name>

<department>2912</department>

</primary - examiner>

</examiners>

</us - bibliographic -data -grant>

</us - patent -grant>

## Excerpt of the RDF representation of a single patent from the year 2005

@prefix patent:<http://us. patents. aksw. org/>.

@prefix patents:<http://us. patents. aksw. org/ontology/>.

@prefix patentp:<http://us. patents. aksw. org/ property/>.

@prefix dc:<http://purl. org/dc/terms/>.

@prefix xsd:<http://www.w3. org/2001/XMLSchema#>.

@prefix foaf:<http://xmlns. com / foaf/0.1/>.

@prefix rdfs:<http://www.w3. org/2000/01/rdf - schema#>.

patent:06906845

a patents: Patent;

patentp: national - classification patent:359290;

patentp: citedBy patent:5285196;

dc: date"2005-06-14"^^xsd: date;

patentp: primaryExaminer patent: examiner - Hung %20 Xuan - Dang;

patentp: agent patent:agent -06906845-01;

patentp: assignee patent: assignee - Samsung Electronics Co., Ltd.;

dc: abstract " The micro - mechanical structure includes an anti - stiction..."@en;

patentp: Year "2005"^^xsd: gYear;

patentp: country patent: country -US;

patentp: applicant patent: applicant -06906845 -001;

patentp: docNo "06906845"^^ xsd:int;

patentp: kind patent:B2;

patentp: inventionTitle "Micro - mechanical device " @en;

patentp: secondaryExaminer patent: examiner -Joseph - Martinez.

patent:359290

patentp: mainSubclassLabel " OPTICAL MODULATOR Light wave temporal modulation..."@en;

patentp: mainClassLabel "Optical: systems and elements" @en;

patentp: mainClassCode "359290".

patent: applicant -06906845-001

a patents: Applicant;

foaf: lastName "Cho" @en;

patentp: country patent: country -KR;

foaf: firstName "Chang -ho" @en;

patentp: city patent: Suwon;

patentp: residence patent: country -KR;

patentp: nationality patent: nationality -KR.

patent: examiner - Hung %20 Xuan - Dang

a patents: Examiner, foaf: Person;

foaf: firstName "Hung Xuan"@en;

foaf: lastName "Dang" @en.

patent:agent -06906845-01

a foaf: Agent;

foaf: Organization "Sughrue Mion, PLLC" @en;

patent: assignee - Samsung Electronics Co., Ltd.

a foaf: Assignee;

patentp: orgname patent: Samsung Electronics Co., Ltd.;

patentp: assignee - city patent: Gyeonggi -do;

patentp: country patent: country -KR;

patentp: role patent:03.

## SPARQL query retrieving the total number of patents per year thus allowing to perform trend analysis.

PREFIX patents:<http://us. patents. aksw. org / ontology/>

PREFIX patentp:<http:// us. patents. aksw. org / property/>

SELECT COUNT (DISTINCT (?patent)) ?year

WHERE

{

?patent a patents: Patent.

?patent patentp: year ? year.

} GROUP BY ? year

## SPARQL query retrieving the number of patents per country and the R&D expenditure as a % of GDP (from the WorldBank dataset).]

PREFIX patentp:<http://us. patents. aksw. org / property/>

PREFIX patents:<http://us. patents. aksw. org / ontology/>

SELECT ?countryPT ?noofpatents ?year ?obsValue

WHERE

{ GRAPH g- indicators: {?observationURI

property: indicator indicator:GB. XPD. RSDV.GD.ZS;

sdmx - dimension : refArea ?refAreaURI;

sdmx - measure: obsValue ?obsValue.}

GRAPH g- meta:

{?refAreaURI a dbo: Country.

?refAreaURI skos: prefLabel ?countryWB.}

SERVICE <http://us. patents. aksw. org / sparql>

{ SELECT COUNT (DISTINCT (?s)) AS ?noofpatents ?countryPT ?year

WHERE{

?patent a patents: Patent.

?patent patent: countrycode ?countrycode.

?countrycode rdfs: label ?countryPT.

?countryPT owl: sameAs ?countryWB.

?patent patentp: year ?year. } } }.

## SPARQL query retrieving the patent citations according to the patent classes/categories.]

PREFIX patents:<http:// us. patents. aksw. org / ontology/>

PREFIX patentp:<http:// us. patents. aksw. org / property/>

SELECT COUNT (?patent) ?citationCategory ?classLabel

WHERE

{

?patent a patents: Patent.

?patent patentp: citedBy ?citing.

?citing patentp: category ?citationCategory.

?patent patentp: national - classification ?nClassification.

?nClassification patentp: mainClassLabel ?classLabel.

} GROUP BY ?citationCategory ?classLabel.
